# Postbiotics: A Promising Approach to Combat Age-Related Diseases

**DOI:** 10.3390/life15081190

**Published:** 2025-07-26

**Authors:** Adel Hamdi, Charmaine Lloyd, Rajaraman Eri, Thi Thu Hao Van

**Affiliations:** School of Science, RMIT University, Bundoora, VIC 3083, Australia; aao8537@hotmail.com (A.H.); charmaine.lloyd@rmit.edu.au (C.L.); rajaraman.eri@rmit.edu.au (R.E.)

**Keywords:** postbiotics, ageing, age-related diseases, antioxidants, gut microbiota, immunomodulation, healthy ageing

## Abstract

Dietary patterns have been identified as one of the most important modifiable risk factors for several non-communicable diseases, inextricably linked to the health span of older people. Poor dietary choices may act as triggers for immune responses such as aggravated inflammatory reactions and oxidative stress contributing to the pathophysiology of several ageing hallmarks. Novel dietary interventions are being explored to restore gut microbiota balance and promote overall health in ageing populations. Probiotics and, most recently, postbiotics, which are products of probiotic fermentation, have been reported to modulate different signalling biomolecules involved in immunity, metabolism, inflammation, and oxidation pathways. This review presents evidence-based literature on the effects of postbiotics in promoting healthy ageing and mitigating various age-related diseases. The development of postbiotic-based therapeutics and diet-based interventions within a personalised microbiota-targeted approach is proposed as a possible direction for improving health in the elderly population. Despite growing evidence, the data regarding their exact mechanistic pathways for antioxidant and immunomodulating activities remain largely unexplored. Expanding our understanding of the mechanistic and chemical determinants of postbiotics could contribute to disease management approaches, as well as the development of and optimisation of biotherapeutics.

## 1. Introduction

Epidemiological evidence suggests a growing expectation of increased human longevity, driven by advancements in public health, medical care, and lifestyle interventions. According to the World Health Organization (WHO), a two-fold increase in the older population demographic has been projected from 2015 to 2050 [1]. Another recent global report predicts that the population of individuals aged 65 and older has increased from 10% since 2022, and will continue to rise to 16% towards 2050 [2]. In Australia, the latest report estimates that 16% of the Australian population were aged 65 years and over, with projections to reach up to 23% by 2066 [3]. These epidemiological shifts are highly correlated to increased longevity, declining birth rates, and lower fertility [1], warranting the need for geriatric health initiatives to support this demographic.

Lifespan, which refers to the duration of life, and healthspan, which refers to the disease-free and functional period, are closely related [4]. A report has estimated a nine-year gap between the two spans [5]. As individuals get older, physiological functions deteriorate due to the accumulation of cellular and molecular damage, a process known as ageing [4]. A balanced interaction between damage and repair, regulated by environmental, stochastic, and genetic factors, has been considered essential for maintaining functions, survival, and ageing rate [6]. Notably, the deleterious effects of ageing increase susceptibility to age-related diseases and contribute to the progressive decline in physical and cognitive function, commonly referred to as frailty [7]. Frailty is generally defined as an increased individual vulnerability to adverse outcomes following exposure to stressors, particularly among individuals of comparable chronological age [8]. Frail individuals experience a decline in quality of life, reduced physical endurance, a higher prevalence of chronic diseases, co-morbidities and disabilities, and increased mortality rates [9]. Ageing and frailty pose significant challenges placing emotional and financial burdens on families and increasing economic pressures due to retirement, altered work patterns, and rising healthcare costs [10,11,12,13,14]. In Australia, the total healthcare expenditure has been predicted to grow at 3.33% annual average rate (from 166 to 320 billion USD) between 2015 and 2035 [15]. Thus, it has become highly imperative to endorse a concept of healthy ageing to deter the predicted unsustainable rise in healthcare expenditures and economic burdens.

The concept of healthy ageing arose from several geroscience-oriented approaches that aim to maintain and develop functional capabilities, promoting well-being in older age [16]. Several multi-dimensional geroscience-oriented interventions, including pharmacological therapy, diet management, and exercises, have shown high translational potential as non-genetic strategies for promoting healthy ageing [17,18]. An example of a current pharmacotherapeutic approach involves the use of the FDA-approved anticancer drug rapamycin, which has been shown to extend healthspan and mitigate undesirable ageing-related immune responses [19,20]. The drug works by inhibiting the mammalian Target of Rapamycin (mTOR) signalling pathway, thus promoting autophagy and protein synthesis, which in turn helps to reduce stem cell exhaustion, mitochondrial dysfunction, and the progression of chronic diseases [21]. Another promising agent is metformin, a therapeutic agent for managing type-II diabetes mellitus, which has also shown potential for prolonging lifespan by slowing the onset of age-related chronic diseases. This hypoglycaemic agent demonstrated lifespan extension in Caenorhabditis elegans, one of the most potent molecular and behavioural ageing model systems [22,23]. Mechanistic aspects of metformin are related to the activation of adenosine monophosphate-activated protein kinase (AMPK) and the inhibition of the target of rapamycin complex (TORC1) [24]. Metformin-based dietary supplements have been shown to improve physical performance, enhance insulin and lipid profiles, and reduce oxidative and pro-inflammatory markers, which promote healthy ageing in mouse models [25]. Though both therapeutic agents (rapamycin and metformin) are promising, their potential to cause gut complications or metabolic impairment may hamper their use in the older population [26,27]. This warrants the need for research for safer biotherapeutics.

Dietary patterns, such as the Mediterranean diet, are recognised as key modifiable factors influencing the onset and progression of age-related diseases, significantly impacting healthspan in older adults [28]. Several nutrient-dense foods, including fruits, vegetables, whole grains, lean proteins, and healthy fats, have been reported to target biomarkers associated with ageing-related pathophysiology and promote healthy ageing [29,30,31]. Despite compelling evidence linking these agents to enhanced health through gut microbiome modulation, the anti-ageing potential of these non-nutrient components warrants further exploration [32,33,34].

**Figure 1:**
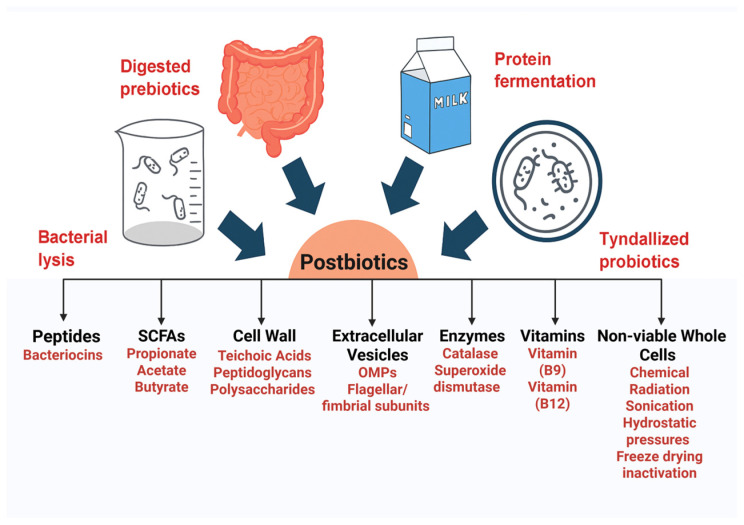
Main postbiotics and their acquisition. Schematic diagram illustrating the main approaches for generating postbiotics. These include bacterial lysis, heat treatment/tyndallisation of probiotic strains, and probiotic-derived fermentation or enzymatic digestion of non-digestible fibres and prebiotics. The resulting postbiotics comprise diverse bioactive compounds, including short-chain fatty acids (SCFAs), polysaccharides, vitamins, extracellular vesicles and more complex components such as proteins, enzymes, and cell wall fragments.

Before introducing postbiotics, it is important to briefly differentiate between the commonly used biotic categories. Probiotics are live microorganisms that provide health benefits to the host when consumed in adequate amounts, though they are sensitive to environmental conditions and may pose safety risks in immunocompromised individuals [35]. Prebiotics are selectively fermented dietary substrates, such as inulin and fructo-oligosaccharides (FOS), that support the growth or activity of beneficial microbes, but excessive intake may lead to gastrointestinal side effects due to osmotic activity [36]. Postbiotics, in contrast, are defined by the International Scientific Association of Probiotics and Prebiotics (ISAPP) as preparations of inanimate microorganisms and their components that offer health benefits to the host [37], offering advantages in safety, stability, and shelf life [38]. These bioactive compounds range from small molecules like hydrogen peroxide to larger ones such as bacteriocins (Figure 1) [39]. They emerge from microbial metabolic activities in the gut, impacting gut health directly or indirectly [40] by producing breakdown of fibrous nutrients known as prebiotics [41]. Postbiotics also encompass bacterial lysates produced through in vitro processes such as Tyndallisation (sterilisation) [42,43,44], and may contain peptides, enzymes, and other complex molecules [41]. These components, often referred to as heat-killed probiotics and metabiotics [45], may also result from in vivo fermentation by-products within the colon, such as branched protein residues, phenolics, short-chain fatty acids, hydrogen sulfide, and indolic compounds [46]. Interest in postbiotics has grown due to their effects on immunity, metabolism, and inflammation [47,48,49,50,51]. It is important to note that the definitions of postbiotics and paraprobiotics remain controversial in the scientific community. While some researchers restrict the term ‘postbiotics’ to purified microbial metabolites [45], others, including the ISAPP consensus, define postbiotics more broadly to include inactivated microbial cells (e.g., heat-killed probiotics) and their components, provided they confer a health benefit to the host. In this review, we adopt the ISAPP definition, which encompasses both inanimate microbial cells and their bioactive constituents [52].

This review aims to provide a comprehensive overview of the types and major bioactive components of postbiotics, along with their mechanisms and health-promoting effects, particularly in the context of healthy ageing. It critically examines current knowledge on their mechanisms of action, including antioxidant activity, immune modulation, and support of gut barrier integrity. Additionally, the review explores the influence of postbiotics on gut microbiota composition, evaluates reported advantages and limitations compared to probiotics, and presents current evidence on their potential role in addressing age-related diseases.

## 2. Key Components of Postbiotics

Several classes of postbiotics have gained significant interest from scientific and industrial communities. In the following section, key postbiotic components will be highlighted, focusing on their roles and proposed mechanisms of action (Figure 1).

### 2.1. Non-Viable Whole Cells

Heat-killing is the most common technique to produce non-viable probiotics, however, other non-thermal approaches, such as chemical inactivation, radiation, magnetic field/sonication, high hydrostatic pressures, and freeze drying, have also been used. Notably, the cellular compositions and bioactivity spectrum of the end product can vary greatly depending on the inactivation method used [53]. For example, bacterial chemical inactivation with glutaraldehyde causes cell death through surface protein cross-linking, altering cellular communication and immune modulation [54]. Inactivation through bacterial cell wall disruption (e.g., sonication) allows relevant host cell interactions with extracted bacterial intracellular components following administration [55]. Either way, non-viable probiotic cells retain many of the biological activities of their live counterparts due to the presence of bioactive components such as enzymes (e.g., superoxide dismutase), peptides, and cell wall-associated molecules. These components can exert effects like scavenging reactive oxygen species, reducing proinflammatory biomarkers, and modulating immunological pathways [56]. Several studies have highlighted the beneficial use of non-viable *Lactobacillus* strains (*L. reuteri* DSMZ-17648, *L. johnsonii*, and *L. acidophilus* LB) in reducing *Helicobacter pylori* load and alleviating symptoms of irritable bowel syndrome [57,58,59].

### 2.2. Bacteriocins

Bacteriocins are ribosomal-synthesised extracellular peptides produced by several lactic acid bacteria, known for their antibiotic-like activity against bacterial pathogens. They play an important role in the nutraceutical industry due to their significant inhibition of food-spoilage pathogenic bacteria such as *Listeria monocytogenes* and *Clostridium difficile* [60]. Two well-established bacteriocins isolated from *Lactobacillus* spp., approved by the US FDA for food preservation and available in commercial forms, are Nisaplin™ (*Lactococcus lactis*) and Alta2341™ (*Pediococcus acidilactici*) [61,62]. The antimicrobial mechanisms of bacteriocins include increasing cell membrane permeability, destabilising the proton motive force, and forming pores within target cell membranes [63]. Their ability to bind with bacterial lipid-II, a precursor for cell wall synthesis, helps prevent the development of bacterial resistance [64]. Combining bacteriocin with several antibiotics provides broad-spectrum coverage against human pathogens, thereby preventing antibiotic abuse and the development of antibiotic-resistant pathogens [65].

Another filed patent (US-9271518B2 and WO-2010117255A1) highlights a combined *Lactobacillus*-derived bacteriocin formulation with potential antimicrobial activity, including reducing *Enterobacteriaceae* faecal counts, increasing *Lactobacillus* counts, improving intestinal membrane integrity, and promoting growth in farming animals [66]. Additionally, a patent for *Lactobacillus casei/paracasei*-derived bacteriocin eye drops (US-20190175670A1) has been filled for strengthening eye epithelial barriers and managing inflammatory eye conditions, such as allergic/infection-origin conjunctivitis and vernal keratoconjunctivitis in both veterinary and human medicines [67].

Bacteriocins also show potential in immunomodulatory activity. In studies on bacteriocin-producing strains of *Lactiplantibacillus plantarum*, researchers found that *L. plantarum* YRL45 effectively reduced elevated levels of IL1β, IL6, TNFα, nitric oxide, and prostaglandin E2 induced by added lipopolysaccharide (LPS) in RAW264.7 cells, thereby alleviating inflammation [68].

### 2.3. Short Chain Fatty Acids

Short-chain fatty acids (SCFAs), mainly acetate, propionate, and butyrate, are produced through the bacterial fermentation of prebiotics and dietary fibres in the human gut [69,70]. These SCFAs are present in human faeces in a typical molar ratio of ~60:20:20, respectively (acetate:propionate:butyrate), and imbalances are associated with health issues [71].

Once absorbed into the bloodstream, SCFAs act as signalling molecules, enhancing insulin function and modulating lipid and glucose metabolism [72]. They regulate liver glucose and lipid levels by downregulating peroxisome proliferator-activated receptor-gamma (PPARγ) [73] and influence immune responses through G-protein coupled receptors (GPCRs) [74]. SCFA-induced GPCR43 modulation supports T-regulatory cell homeostasis and suppresses pathogenic bacteria like *Prevotellaceae* and *Helicobacter* spp., maintaining colon health [75]. Studies highlighted the preservation of the gut membrane barrier and the reversal of hepatic ischemia through butyrate’s action in preventing endotoxin translocations, macrophage activation, amplified CD103^+^ dendritic cells actions, and restored tight junction protein Zonula occludens-1 [76,77].

### 2.4. Extracellular Vesicles

Scientific interest has grown in probiotics’ extracellular vesicles as promising postbiotics with potential clinical applications [78]. These vesicles contain enzymes for peptidoglycan degradation (lysozyme-like or murein hydrolases), adhesion molecules (fimbrial/flagellar subunits; FOCs/FLIs), and other membrane proteins (OMPs) [79,80]. Several in vivo and in vitro studies have highlighted their immunomodulation, anti-inflammatory, and gut barrier-regenerative activities [78]. Extracellular vesicles from strains such as *Escherichia coli* Nissle-1917 [81], *E. coli* O6K5H-1 [82,83], *Akkermansia muciniphila* ATCCBAA-835 [84,85,86], *L. plantarum* WCFS-1 [87], *L. plantarum* Q7 [88], *Lentilactobacillus kefirgranum* PRCC1301 [89], and *Clostridium butyricum* MIYAIRI-588 [90,91] have shown protection of the intestinal barrier against pathogens, microbiota regulation, and related immunomodulation. Notably, a study showed skin anti-ageing activity including improved elasticity, increased water content, and reduced skin pigmentation/wrinkles in Korean women following topical applications of *L. plantarum*-derived extracellular vesicles [92].

### 2.5. Peptidoglycans as Bacterial Cell Wall-Derived Postbiotics

Peptidoglycans isolated from probiotic microorganisms have demonstrated immunomodulation, anti-inflammatory, and anti-cancer activities [53,93,94]. Anti-inflammatory activity of *L. casei*-derived peptidoglycans is seen through the downregulation of IL-12 [95]. Administration of muramyl dipeptide-linked peptidoglycans has inhibited adipocyte inflammation and reduced glucose intolerance in obese animal models without causing weight gain or altering gut microbiota [96]. Soluble proteins with peptidoglycan hydrolase activity (p40/75) secreted by the Gram-positive *L. rhamnosus* GG have shown significant protective effects on gut lining integrity while suppressing intestinal cell apoptosis and promoting epithelial homeostasis [97].

### 2.6. Teichoic Acids

Teichoic acids are one of the important structural components for the integrity and function of the bacterial peptidoglycan cell wall, especially in Gram-positive bacteria, playing roles in adhesion and bacteria-host signalling [98,99,100,101]. They can trigger host immune responses, leading to the release of cytokines such as IL-1β, -6, -8, -12, and IFN-α/TNF-α [102]. The immunomodulating activity of teichoic acid postbiotics has been demonstrated with co-administration of 5-fluorouracil and *Bifidobacterium*-derived teichoic acids [103]. The anti-inflammatory activity of *L. paracasei*-derived teichoic acids is shown through increased phospho-p38-AMPK levels and suppression of pro-inflammatory NF-κB signalling, reducing cytokine production [104]. Conversely, teichoic acids from pathogenic bacteria like *Staphylococcus aureus* have been observed to induce excessive inflammation, contributing to gut barrier deterioration [105,106].

### 2.7. Polysaccharides

Polysaccharides are sugar molecules linked by glycosidic bond that are involved in the structure of several bacterial cell wall components. Several pre-clinical reports demonstrated the wide-range activity of polysaccharides as postbiotics, exerting anti-inflammatory, antioxidant, and immunomodulatory effects [107]. Exopolysaccharides from a non-pathogenic strain of *Bacillus* sp. (LBP-32) showed potent antioxidant activity in inflammation-induced RAW264.7 macrophages, primarily through downregulating reactive oxygen species (ROS) accumulation [108]. These antioxidant effects were linked to the inhibition of NF-κB/mitogen-activated protein kinase signalling.

Exopolysaccharides from *Lactobacillus delbrueckii* exhibited strong in vivo immunomodulatory effects by enhancing IgG, IgA, and IgM production; demonstrated potent in vitro antioxidant activity; showed antimicrobial effects against *Bacillus subtilis* and *S. aureus*; and increased red blood cells (RBC), white blood cells (WBC), and packed cell volume levels in tumour-induced mice [109]. Purified exopolysaccharides from *Lactobacillus fermentum* S1 also showed strong antioxidant and antimicrobial activities, and improved gut transit tolerances [110]. Administration of exopolysaccharides from *Lactobacillus buchneri* TCP016 modulated gut microbiota composition, benefiting the attenuation of inflammation-induced liver injuries [111].

### 2.8. Enzymes

Bacteria can release enzymes, many of which have antioxidant potential [112]. Cell-free extracts of *Streptococcus thermophilus* and *L. delbrueckii* demonstrated high levels of antioxidants, such as superoxide dismutase [113]. Administration of catalase from *L. lactis* increased oxidative tolerance and reduced intestinal injury severity in 1,2-dimethylhydrazine-induced colon cancer mice. Tomusiak-Plebanek et al. highlighted that the anti-inflammatory activities of several *Lactobacillus* strains in inflammatory bowel disease mice models are strongly associated with antioxidant enzyme expression levels [114]. Additionally, several *Bifidobacterium* strains showed hydrogen peroxide degradation capability due to their NADH-peroxidase enzyme content [115].

### 2.9. Vitamins

Vitamins are essential enzyme co-factors involved in regulating numerous human physiological processes and maintaining health. They are primarily obtained from the diet, but some can also be synthesised by bacterial processes [116]. Folates contribute to indirect antioxidant activity by enhancing tetrahydrobiopterin absorption, which facilitates the elimination of ROS [117]. Several probiotic strains, particularly those within the genus *Bifidobacterium*, have been identified as folate (vitamin B9) producers, a vital nutrient for DNA synthesis and repair. Among them, *B. adolescentis* and *B. pseudocatenulatum* are among the most efficient folate-producing strains in both in vitro and in vivo studies [118]. Vitamin accumulation has been reported in *Bifidobacterium* strains, where elevated folic acid levels were observed [119]. Cyanocobalamin (vitamin B12), which is important in dairy products, is recognised for its role in free radical scavenging and glutathione regeneration [120]. Genomic analyses of several *Lactobacillus* strains have revealed the presence of genes encoding enzymes required for cyanocobalamin biosynthesis [121].

## 3. Advantages of Postbiotics over Probiotics

This postbiotics offer several advantages over live probiotic bacteria due to their direct and indirect positive healthcare benefits and specific characteristics. Unlike probiotics, postbiotics exhibit consistent dose standardisation, reduced variability in effectiveness, and greater tolerance to gastric juices, bile, and proteolytic enzymes [122,123]. They also offer logistic benefits such as feasible storage, simpler transportation, maintenance, and extended shelf-life (up to five years) [124]. Postbiotics support rapid production technologies and precise quantitative control approaches [38]. Postbiotics are more suitable for special populations, such as infants and children, promoting gut health during early development, aiding immune system maturation and reestablishing the Th1/Th2 balance [125]. The safety profile of postbiotics is superior to probiotics, mimicking probiotic benefits without the risks of administering live microbes, which may carry virulence factors or acquire antibiotic resistance genes during colonisation in vulnerable individuals [38]. Probiotics have been associated with adverse effects such as bloating, flatulence, bacteraemia, fungemia, and antibiotic resistance gene transfer, especially in the elderly, immunosuppressed, and those with compromised intestinal barriers [35]. Consequently, postbiotics are emerging as a safer alternative for promoting healthy ageing and treating age-related diseases. This will be the main focus of the presented review.

## 4. The Role of Postbiotics in Healthy Ageing and Age-Related Diseases

### 4.1. Gut Microbiota Dysbiosis as a Hallmark of Ageing and the Role of Postbiotics in Healthy Ageing

**Figure 2:**
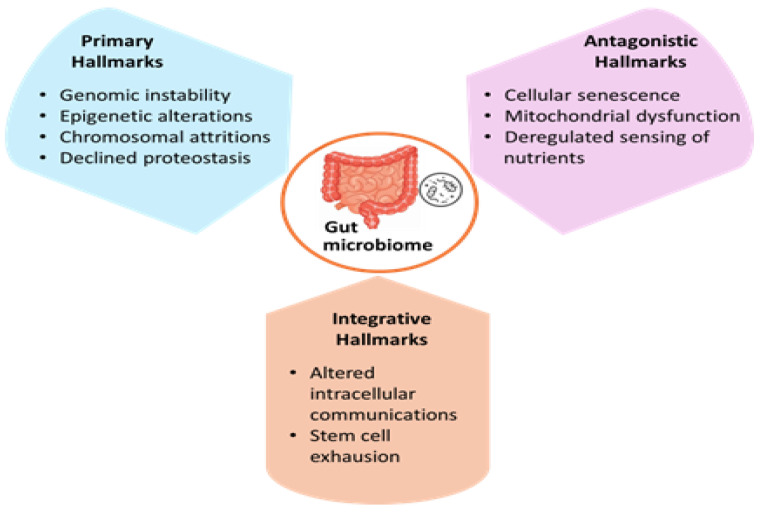
Hallmarks of aging and their interconnection with the gut microbiome. Nine hallmarks of aging are identified and categorised into three main classes: (i) primary hallmarks, (ii) antagonistic hallmarks, and (iii) integrative hallmarks. Gut microbiome dysbiosis and altered microbial diversity have emerged as central elements, closely interconnected with other aging hallmarks due to their significant impact on host physiological functions.

Recently, growing evidence has highlighted gut dysbiosis and altered microbiome diversity as central hallmarks of ageing [126,127,128]. Initially, various hallmarks of ageing were outlined and categorised into three classes: (i) primary damaging hallmarks, (ii) antagonistic hallmarks in response to primary injury, and (iii) integrated hallmarks that arose due to the interaction between both previous groups. The specific biological processes corresponding to primary, antagonistic, and integrative hallmarks are summarised in Figure 2 [129].

Damaging hallmarks include hampering of immune surveillance and promoting of immunosenescence to accelerate the accumulation of senescent cells, tissue fibrosis, organ dysfunction, and eventually unhealthy ageing, making individuals more susceptible to disease [130]. The interconnection between triggered immune responses, aggravated inflammation, and oxidative stresses has also been postulated to be contribute with the pathophysiology of primary damaging hallmarks [129]. Antagonistic hallmarks include gut microbiota dysbiosis, triggered inflammatory pathways, and pathophysiological cascades correlated to unhealthy ageing [131,132,133]. Studies on the metagenomic analysis of elderly subjects’ gut microbiome revealed a downregulation in the expression of genes associated with short-chain fatty acid (SCFA) production, upregulation of tryptophan/proteolytic metabolic pathway genes, and a preferential shift towards putrefactive metabolism leading to pathological inflammations [134,135]. Additionally, the gut microbiota has been shown to influence the duration of a healthy lifespan, as demonstrated by a study in which recolonising the gut of middle-aged killifish with microbiota from younger fish extended the lifespan of the recolonised animals [136]. Another study highlighted the impact of microbiota transplant from wildtype to progeroid (prematurely aged) rodents, where healthier gut functioning was promoted in the latter animals. These recipient rodents exhibited more diverse gut microbiota with higher production of short-chain fatty acid genera [137]. Based on the above evidence, postbiotics have emerged as potential tools for promoting healthy ageing by being able to modulate gut microbiota [138,139,140] and impart immunomodulation properties [47,141,142].

### 4.2. Current Understanding of Postbiotic Mechanism of Action

#### 4.2.1. Antioxidant Effects and Oxidative Stress Mitigation

Antioxidant activity has been one of the principal areas of interest for researchers aiming to combat cellular damage and injury [143]. Scavenging free radicals from damaging cell membranes, DNA, protein, and other cell components has been a beneficial strategy to mitigating many diseases and promoting healthy ageing, as oxidative stress plays a central pathophysiological role in many conditions [144]. Generally, non-pathogenic microorganisms including probiotics have developed antioxidant strategies against harmful ROS (Table 1)*,* such as producing several antioxidant enzymes like superoxide dismutases, glutathione peroxidases, NADH-oxidases, and catalases [38].

Notably, several probiotics belonging to the *Bifidobacterium* species (*B. longum*, *B. infantis*, *B. adolescentis*, and *B. breve*) have been reported to produce significant amounts of NADH peroxidase, which degrades the hydrogen peroxide [155]. Thus, enzymatic postbiotics have been considered promising, as *L. plantarum* cell-free supernatant in in vivo models was correlated to high glutathione peroxidases content [146,147]. Cellular extract postbiotics from *L. casei* also showed higher antioxidant activity compared to other species in in vitro free radical scavenging tests [156]. Exopolysaccharide postbiotics secreted by several *Lactobacillus* strains and *Paenibacillus mucilaginosus* TKU032 have emerged as potential antioxidant agents with radical scavenging capabilities [110,150]. Elevated levels of uronic acid have been suggested by several studies to correlate with antioxidant capacity of *Lactobacillus helveticus* MB2-1 and *Bifidobacterium animalis* RH [151]. Uronic acid, a negatively charged polysaccharide, has been shown to possess iron-chelating properties that inhibit ROS production, thereby preventing the formation of hydroxyl radicals [157].

#### 4.2.2. Anti-Inflammatory and Immunomodulatory Properties

Disruptions in gut microbiota homeostasis have been linked to overstimulation of inflammatory responses, contributing to chronic illness through downregulated anti-inflammatory signalling mechanisms [158]. Studies have highlighted the capability of postbiotics to mitigate pro-inflammatory signalling and reduce cytokine storm production, making them promising candidates for adjuvant therapy in conditions like SARS-CoV-2 [159].

Research on *B. longum* CECT7347-derived postbiotics showed prominent anti-inflammatory activity and reduced gut disturbances through specific immune responses [145]. Another study showed increased levels of IL-6 and IL-1β in porcine immunocytes stimulated by *L. lactis*-derived bacteriocins, which mediated T-/B-cell differentiation and proliferation [160]. Bacteriocins, which are extracellular peptides produced by various lactic acid bacteria, have been linked to significant anti-inflammatory activity [161,162]. Other bacteriocins were reported to halt inflammations induced by intestinal and urinogenital infections [163].

The interest in postbiotics for modulating immune responses arose following findings by Tejada-Simon et al., who used inactivated probiotics and cellular components to activate leukocyte immune lineage [164]. Several studies have suggested a potential interlink between postbiotics’ cellular components and immune response modulation through an association with innate immune system sensors (Table 2) [165,166].

Specialised pattern recognition receptors (PRRs), including nucleotide-binding domain (NOD)-like receptors and toll-like receptors (TLRs), are typically abundant on host immune cells and responsible for recognising and interacting with pathogen’s cellular components [171]. The protein/glycoprotein self-assembly subunits known as surface(S)-layer were also highlighted for triggering host immune responses [172]. A study by Konstantinov et al. demonstrated that *L. acidophilus*-purified S-layers specifically bind to cellular C-type receptors on host dendritic cells and macrophages [173].

Moving towards bacterial-secreted postbiotics, exopolysaccharides have been highlighted as immuno-activating macromolecules that act as microbe-associated molecular patterns, capable of binding to pattern recognition receptors (PRRs) [174]. Zhou et al. showed that presenting exopolysaccharides as antigens via CD103+ dendritic cells promotes chemotaxis and lymphoid tissue recruitment through secreted cytokines [175]. These exopolysaccharide postbiotics were shown to increase interferon levels, upregulate TLR-3 expression, and enhancing chemokine responses, including CC motif ligand-4 and CXC motif chemokine-10 [169,176,177]. Another study found that the probiotic strain *S. thermophilus CRL1190* and its exopolysaccharides reduce *H. pylori* adhesion and inhibit the inflammatory response in human gastric adenocarcinoma epithelial cells.

Short-chain fatty acids have been shown to interact with their recognised receptors (free-fatty acid G-protein coupled receptors) to maintain the homeostasis of intestinal T-regulatory lymphocytes [75]. This is demonstrated by promoting the growth of gut commensal *Bifidobacterium* spp. while inhibiting pathogenic bacteria such as Prevotellaceae and *Helicobacter* spp. [75]. Activating CD103+ dendritic cells through fibre-rich diets have been illustrated to stimulate SCFA production, maintain healthy gut microbiome, and protecting the intestine against food allergies [76]. Other probiotic metabolites, like lactic acid and indole derivatives, have been demonstrated to impact the immune responses. Intestinal CX3CR1+ immune cells extend dendrites in response to lactic acid in a GPR-31- dependent manner [178].

From a comparative viewpoint, several studies demonstrated the superior immunomodulatory activity of postbiotics derived from inactivated microorganisms over live probiotics. Notably, heating inactivation of probiotics was found releasing several heat shock proteins with enhanced immunomodulatory effects [179]. In a study comparing live and heat-killed *L. casei* Zhang (LcZ), both forms were shown to improve the innate immune response of macrophages by enhancing the expression of pro-inflammatory cytokines and the transcription of Toll-like receptors, with the heat-killed form offering potential benefits in terms of safety for immunocompromised individuals [180]. Comparative findings between the inactivated and live versions of different *Bacillus* spp. (*B. subtilis* FPTB13 and *B. amyloliquefaciens* FPTB16) reported higher cellular-based immune responses with the inactivated species [181]. Another study showed that heat-inactivated *Lactobacillus salivarius* A6, *Lactobacillus gasseri* A5, and *L. acidophilus* A2 postbiotics were capable of modulating Th1-driven immune responses by triggering splenocyte interferon-γ production, IL-12/-10 p70 proliferations, and dendritic cell-based IL-12 p70 secretion [182].

#### 4.2.3. Postbiotics and Gut Barrier Integrity: Mechanisms and Protective Role

Maintaining gut barrier integrity is important for human health, as it protects against pathogens and allergens, while preserving the balance between the immune system and commensal microbes. Important components of gut barrier include intestinal layers, microbiota, immunocytes, antimicrobial proteins, and tight junction peptides [183]. Studies have highlighted the role of postbiotics in fortifying the gut barrier similar to live probiotics. The general mechanisms involve: (i) forming a physical barrier against pathogen adherence to the gut membrane, (ii) immunomodulation, (iii) microbiota modulation, and/or (iv) direct antimicrobial activity [48,138].

Administrating heat-inactivated *L. rhamnosus* in colitis mice models showed protective mucosal permeability and barrier function restorations, which were suggested to be correlated with upregulated intestinal zonula occludens-1 and myosin light-chain kinases [184]. *L. paracasei*-derived postbiotics showed enhanced mucin-2 expressions in a constipation animal model, helping to restore the gut barrier functions [185]. Another in vivo study demonstrated the upregulation of tight junction peptides in a colitis mice model following administration of lipoteichoic acid from *L. fermentum* MTCC5689, *L. plantarum* MTCC5690, or *L. rhamnosus* GG [186].

Increased tight junction peptide expressions, especially claudin-1/4 and occluding, was also observed in post-weaning lambs and nematode models following supplementation with *L. plantarum* [187] or heat-inactivated *B. longum* CECT7347 [145]. Supernatant isolated from *L. rhamnosus* GG has been shown to aid in protecting human intestinal smooth muscle from injuries and cellular damage [188]. In *E. coli*-treated Caco-Goblet cells, serving as a model for gut cellular insult, the combined antidiarrheal agent (gelatine tannate) with inactivated *Lactobacillus* strains (Tasectan Duo) showed synergistic protection by decreasing paracellular fluxes and increasing transepithelial electrical resistances compared to the other test compounds [189].

SCFAs like butyrate were associated with supportive regeneration of the gut epithelium [190]. Possible mechanistic aspects of SCFAs involve modulating Caco-2 cell trans-permeability, enhancing transepithelial electrical resistances, and elevating gene expressions of tight junction proteins [191,192].

## 5. Gut Microbiome Signatures in Ageing and the Role of Postbiotics in Age-Related Disease Mitigation

The gut microbiota is a diverse and complex microbial community that co-exists within the gut of multi-cellular hosts, shaped by the host’s habitual diets, behaviours, as well as social and environmental factors [193]. This microbial community can consist of more than 2000 microbial species, depending on host-associated factors. The gut microbiota exhibits host specificity due to its composition fingerprint [194].

Several studies highlighted the diverse role of gut microbiota in host physiology including metabolic, anti-inflammatory/immunomodulating, and neurological actions [195]. The metabolic advantages of these commensal microbes stem from their capability to utilise organic and inorganic compounds and catabolise plant-based foods [196]. These microorganisms participate in the host’s carbohydrate, fat, and protein metabolic pathways, thus impacting the host’s physiological health status and susceptibility to metabolic disorders [197,198,199].

Anti-inflammatory/immunomodulation effects of the gut microbiome result from the interactions of microbial metabolic products and surface molecules with the host’s signalling receptors [200,201]. Regarding neurological impacts, evidence has highlighted the role of specific gut microbiota in reducing stress and improving cognitive and memory functions [202]. Studies further link these positive neurological impacts to the microbiome-derived metabolites that modulate immunoinflammatory responses and neuronal plasticity against inflammation and oxidation injuries [203].

### 5.1. Age-Related Changes in Gut Microbiome Signatures and Their Health Implications

Several factors, both internal and external, have been reported to impact the quantity and composition of the gut microbiome. These influencing factors can generally be categorised into social, physiological, and health status/disease conditions [195]. Deteriorations in the gut physiology highly influences gut microbiota. This deterioration generally progresses with ageing due to increased inflammation, genomic instability, decreased proteostasis, epigenetic dysregulation, and cellular dysfunction [204].

External factors such as poor lifestyle, diet quality, physical inactivity, and medications can also negatively affect the gut microbiome [205]. However, detailed studies investigating these external factors influences on gut microbiome signatures remain limited. Social networking is also another influencing factor for gut microbiome composition, as individuals in the same residences often exhibit comparable gut microbiome communities [206].

### 5.2. Microbiome Signatures in Healthy vs. Unhealthy Ageing

Several studies have identified general age-related gut microbiome signatures. With chronological ageing, there is a significant loss of dominant beneficial microbiome taxa, including the *Coprococcus* sp., *Faecalibacterium* sp., *Roseburia* sp., *Prevotella* sp., *Lachnospira* sp., and *Eubacterium rectale*, as well as the health-related genera *Lactobacillus* sp. and *Bifidobacterium* sp. (Group 1) [205,207]. This first group of commensal microorganisms seems to be replaced by other commensal taxa such as the putative-advantageous genera *Odoribacter* sp., *Barnesiella* sp., *Butyricimonas* sp., *Butyricicoccus* sp., *Akkermansia* sp., *Oscillospira* sp., and *Christensenellaceae* (Group 2), as well as pathological taxa, *Anaerotruncus* sp., *Streptococcus* sp., *Bilophila* sp., *Eggerthella* sp., *Enterobacteriaceae*, *Escherichia* sp., *Desulfovibrio* sp., and *Fusobacteria* (Group 3) [207]. These later gut microbiome alterations represent a definitive signature for ageing, particularly unhealthy ageing [208].

Comparative studies on gut microbiome signatures in healthy ageing (characterised by longevity, high physical activity, healthy diets, and/or low cognitive decline) versus unhealthy ageing (frailty, inflammation, cognitive disorders, cardiovascular disorders, obesity, reduced physical activity, migraine, metabolic syndrome, osteoporosis, and pre-mortality) have shown overlapping findings [209,210,211,212,213]. Unhealthy ageing is marked by the loss of dominant commensal taxa, similar to chronological ageing, along with the acquisition of wider-range pathological taxa, including Group 3 plus disease-associated *Clostridium* spp. (*C. clostridioforme*, *C. hathewayi*, *C. symbiosum*, *C. citroniae*, and *C. bolteae*) and *Ruminococcus torques* [209,214]. In contrast, the healthy ageing group exhibited a gut microbiome signature similar to that of Group 2, with an increase in commensal taxa that rise during chronological ageing but decline in age-related disorders of unhealthy ageing. This group includes *Odoribacter, Barnesiella, Butyricimonas*, *Butyricicoccus*, *Akkermansia*, *Oscillospira*, and *Christensenellaceae*, which are considered markers of healthy ageing [211,215].

In brief, the gut microbiome shifts with chronological ageing and the host’s physiological conditions. Group 1 taxa are typically replaced during chronological ageing and especially throughout unhealthy ageing. Group 2 taxa represent healthy ageing-related microbiome signatures that increase with chronological ageing but decline during age-related health deterioration. Finally, Group 3 are the pathological taxa that become more abundant with ageing, particularly in unhealthy ageing conditions [204].

### 5.3. Clinical Evidence of Postbiotics in Modulating Gut Microbiota and Promoting Healthy Ageing

Research has highlighted the role of postbiotics in stimulating the growth and functional activities of various gut microbiota when beneficial bacteria (e.g., *Bifidobacterium* and *Lactobacillus* spp.) are insufficient [216]. By enhancing probiotics, postbiotics offer potential health benefits in maintaining gut eubiosis, preventing pathogen colonisation, preserving intestine wall integrity, and enhancing a balanced host-microbiome relationship [217]. Furthermore, postbiotics can alter gut microbiome compositions towards specific taxa, thereby improving the gut’s physiological status [122].

A study by Zhang et al. demonstrated strong modulation for faecal microbiota composition, beta diversity, and metagenomic potential in mice with colitis following administration of *Bifidobacterium adolescentis* B8589-derived postbiotics as compared to the live parent probiotic [138]. The authors reported a marked increase in *Muribaculaceae* spp., *Bacteroidales bacterium M9*, *Bacteroidales bacterium M10*, and *A. muciniphila*, while observing negative correlations with *E. coli*, *Bacteroides intestinalis,* and *Helicobacter bilis* in the postbiotic-fed mice groups. The gut microbiome composition findings were aligned closely at the phylum level with other studies on the faecal microbiome [90,218,219,220]. Another study highlighted species-level gut microbiota changes following postbiotic administrations [221]. This evidence highlights the potential development of postbiotic-based therapeutics derived from healthy signature bacteria and the promotion of personalised microbiota-targeted interventions to enhance healthy ageing and longevity in the elderly population. Further longitudinal studies are needed to confirm roles of signature bacteria and their postbiotics for the elderly.

## 6. Specific Age-Related Diseases and Postbiotic Interventions

**Figure 3:**
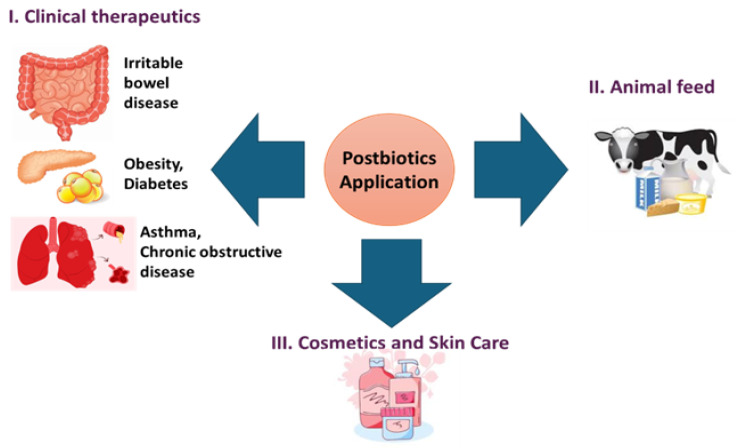
Applications of postbiotics in clinical therapeutics, agricultural animal feed, and cosmetic skincare.

Postbiotics exhibit great potential for a wide-range of therapeutic and industrial applications, improving the health and personal care of both humans and animals (Figure 3) [183].

To alleviate respiratory disorders and reduce recurrent episodes, bacterial lysates from a wide range of pathogenic bacteria (polyvalent mechanical bacterial lysate) and Lantigen B bacterial lysate derived from *Streptococcus pneumoniae* type-III, *Streptococcus pyogenes* group-A, *Branhamella catarrhalis*, *S. aureus*, and *Hemophilus influenza* have showed beneficial effects [222,223]. Food supplements containing heat inactivated *Mycobacterium manresensis* have been effective in managing latent tuberculosis through T-regulatory lymphocyte regulation [224]. While these bacterial lysates are classified as postbiotics due to their non-viable microbial origin and health-promoting effects, they also share structural and functional similarities with oral killed vaccines, particularly in their immunostimulatory role.

Furthermore, several postbiotics have shown promise in modulating immune checkpoint inhibitors, enhancing their anticancer activity [225]. Clinical trials of combined oral administration of *L. rhamnosus* with immune checkpoint inhibitors (such as programmed cell death protein-1 inhibitors) provided better prognosis for non-small cell lung cancer compared to monotherapy [226]. Additionally, *L. bulgaricus*-derived lysozyme-based lysates reduced chemotherapeutic adverse effects in leukemia patients by supporting WBC regeneration and enhancing the immune system [227]. Focusing on age-related diseases, the detailed role of postbiotic interventions is thoroughly described in the following context.

### 6.1. Cardiovascular Diseases and Metabolic Disorders

Older adults face an increased risk of cardiovascular diseases, as aging itself serves as an independent risk factor. Nevertheless, this risk factor has been also compounded by other additional comorbidity factors, such as diabetes, frailty, and obesity [228]. These medical conditions promote pro-inflammatory and pro-thrombotic states, along with elevated blood pressure, atherogenic hyperlipidemia, and hyperglycemia [229]. Besides the progression of metabolic disease, the host’s ability to utilise energy from food has been linked to gut dysbiosis. In this context, postbiotics have shown promise in alleviating metabolic disorder-related factors without significantly altering the gut microbiome composition [230]. This is particularly relevant given that hypertensive patients often exhibit reduced levels of beneficial gut bacteria, including *Oscillibacter*, *Faecalibacterium*, *Bifidobacterium*, *Roseburia*, *Butyrivibrio*, and *Coprococcus*, compared to healthy individuals, a microbial shift associated with inflammation and metabolic imbalance [231].

Postbiotics, particularly SCFAs, are the most recognised metabolites impacting blood pressure through various mechanisms. They exhibit anti-inflammatory, immunomodulatory and antioxidant properties [232], and inhibit hepatic lipid synthetases while reducing uremic toxin biolevels, both of which contribute to maintaining vascular integrity [233,234]. The role of postbiotic SCFAs in blood pressure regulation is suggested by their ability to diminish the angiotensin II-driven hypertension in mouse models following butyric acid administration [235]. Propionic and acetic acids, but not butyric acid, showed affinity for the human olfactory G-protein coupled receptor (GPCR; Olf78/OR51E2), which influences blood pressure modulation by triggering renin secretion from juxtaglomerular cells. This confirms the potential of these fatty acids in regulating vascular functions [236].

On the other hand, the three SCFAs activated mouse G-protein-coupled receptors (GPCRs): GPR41 (free fatty acid receptor; FFA3), GPCR43 (FFA2), and GPCR109a, which in turn reduces NF-κB activation and consequent pro-inflammatory mediator (TNF-α, IL-1β, and IL-6) productions [237]. GPCR43 imposes indirect impacts on blood pressure by regulating inflammatory pathways, influencing blood-brain barrier permeability and increasing glucagon-like peptide production [238]. A summary of preclinical and clinical evidence for SCFAs in cardiovascular diseases is illustrated in Table 3.

Besides short-chain fatty acids, other postbiotic components, including exopolysaccharides and lipoteichoic acids, have been highlighted for their potential cardiovascular activities [247]. Another study found comparable antioxidant effects in plasma and liver samples of an in vivo mice model following oral administration of *L. lactis*-derived exopolysaccharides [248]. A multi-pharmacological activity profile has been demonstrated with *Lactobacillus kefiranofaciens*-derived exopolysaccharides (Kefiran) where blood pressure elevation and serum lipid contents were reduced in stroke-prone spontaneously hypertensive rats [249]. Regarding lipoteichoic acids, anti-atherosclerotic effects through immunomodulation/anti-inflammation have been highlighted for this postbiotic in both in vitro and in vivo experiments. Treatment with *L. plantarum*-isolated lipoteichoic acids reduced IL-8/TNF-α expressions in human leukemic monocytes (THP1) cells as well as up to 60% THP1-Human umbilical vein endothelial cell (HUVEC) inhibited adhesion capability owing to reduced adhesion molecule expressions; VCAM1, ICAM1, and E-selectin [250]. Moreover, parenteral administration of *L. plantarum*-isolated lipoteichoic acids to high-fat/cholesterol diet animal model showed reduced macrophage/monocyte infiltrations towards the arterial lumen [250].

Postbiotics can also indirectly impact cardiovascular disease risks by altering metabolic abnormality covariates including hyperglycaemia and obesity. Activation of GPCR43 via short-chained fatty acids has been reported to inhibit insulin-driven lipid accumulation within the white adipocytes and regulate energy homeostasis through lipid consumption promotion [251]. Intestinal GPCR43 and GPCR41 activation has been also correlated with satiety and glucose homeostasis [252]. The GPCR-fatty acid interaction was depicted through stimulated secretions of glucagon-like peptide-1 and peptide YY from intestinal endocrine L-cells [251]. Both butyrate and propionate have been shown to improve glucose/energy homeostasis through sympathetic neuron activation and the promotion of intestinal gluconeogenesis [253]. Notably, *B. animalis* IPLA R1-isolated exopolysaccharides showed a positive impact on insulin sensitivity, as oral administration in high-fat diet-fed rats reduced serum insulin levels without causing significant fluctuations in homeostasis model assessment scores or fasting glucose indices [254].

### 6.2. Neurodegenerative Disorders

Preclinical investigations have demonstrated a positive correlation between several probiotic secretomes and age-associated biological processes related to the host’s central nervous system (CNS) functioning, including system senescence and neurodegenerative disease pathogenesis [255,256,257]. In addition to their immunomodulation, anti-inflammatory and antioxidant properties relevant to neurodegenerative diseases, postbiotics can positively impact blood-brain barrier integrity, which is a key therapeutic target in disease treatment [258]. Evidence has shown that the depletion of SCFAs following long-term antibiotic-induced gut dysbiosis is associated with increased CNS barrier permeability and mood/cognitive impairment in animal models [259]. The neuroprotective mechanisms of supernatants isolated from *Megasphaera massiliensis* and *Parabacteroides distasonis* relevant to Alzheimer’s and Parkinson’s pathology have been highlighted through their antioxidant properties, along with promoting neural cell differentiation and reducing microglia-derived cytokine production [260]. Fermentation by *L. plantarum* and *B. longum* showed increased neuron viability, boosted brain-derived neurotrophic factor (BDNF) expression, and reduced pro-apoptotic gene expression [261,262].

The interaction of SCFAs with their molecular host receptors has been reported to be crucial for neurogenesis in both wildtype and Alzheimer’s animal models [263], and they have been shown to elevate serum levels of brain-derived neurotrophic factors in patients with attention deficit hyperactivity disorder (ADHD) [264]. Propionate and butyrate isolated from *Lactobacillus* species have been linked to restored blood-brain barrier homeostasis and integrity following LPS-induced CNS injury in animal models [265,266]. Exopolysaccharides from different *Lactobacillus* strains also exhibit antioxidant activities by protecting PC-12 and β-amyloid-induced SH-SY5Y cell injury against oxidative stresses [267,268]. *L. plantarum*-derived extracellular vesicles upregulated brain-derived neurotrophic factor (BDNF) expression and alleviated stress-triggered depressive-like behaviours in a cortisone-induced stress model using a mouse hippocampal cell line and in vivo restraint-stressed mice [269]. Furthermore, extracellular vesicles from *L. rhamnosus* ameliorated inflammatory responses in lipopolysaccharide-treated microglia in mice [270]. Protection of in vitro mice hippocampus-derived HT22 cells and in vivo Tg-APP/PS1 mice against β-amyloid induced oxidative stress were highlighted for these postbiotics [271].

### 6.3. Bone Health and Osteoporosis

The elderly population is at significant risk of osteoporotic fractures which are associated with poor life quality, morbidity, and even mortality [272]. Bone homeostasis is regulated by a gut-immune axis where the gut microbiome promotes the development of cellular regulatory B- and T-lymphocyte and Th1/2 immune cells, preventing the formation of osteoclast (bone resorption cells) via IL-4/10, cytotoxic T-lymphocyte–associated antigen-4, transforming growth factor-β, and interferon-γ [273,274]. Furthermore, transforming growth factor-β secreted by regulatory T-/B-cells promotes osteoclast (bone-forming cells) differentiation, making the gut microbiome essential in modulating the balance between bone formation and resorption balance [275].

Notably, evidence from the literature highlights several postbiotics that exhibit anti-osteoporotic activities [276]. Postbiotics isolated from *L. plantarum* MD35 were shown to impact osteoclast differentiations at mouse bone marrow-derived macrophage cultures through attenuated signal-related kinase phosphorylation and suppressed NF-κ-mediated osteoclast-related gene expression [277]. Additionally, in vivo, findings highlighted the effective protective activity of *L. plantarum* MD35-postbiotic against estrogen-deficient-triggered bone loss in a postmenopausal osteoporosis mouse model. Isolated SCFAs played a significant role in bone homeostasis regulation by downregulating osteoclast-related bone resorption and sustained T-reg lymphocyte-driven inflammatory reactions [278].

Further, postbiotics isolated from *Lactobacillus curvatus* 38-CS- were reported to inhibit NF-κ-mediated osteoclast differentiation and bone loss in a postmenopausal osteoporotic mouse model through the downregulation of TRAF6/NF-κB/MAPKs axis [279]. Suppression of osteoclastogenesis has been observed with cell supernatant postbiotics derived from *L. rhamnosus*, *L. reuteri*-6475, and *Lactobacillus salivarius* MG4265 by modulating the immune system and/or NF-κ-mediated osteoclast-related gene expressions [275,280,281]. Propionate and butyrate supplementation in high-fibre diet mice showed increased bone masses through reprogramming osteoclast metabolism (shifting from oxidative phosphorylation to enhanced glycolysis) and downregulation of osteoclast-associated gene (NFATc1 and TRAF6) [282].

## 7. Economic Potential and Regulatory Outlook of Postbiotics

The economic potential of postbiotics is increasingly recognised in both the food and pharmaceutical sectors, driven by growing consumer demand for safe, stable, and effective microbiome-targeted products [283,284]. Their incorporation into functional foods, such as dairy and non-dairy beverages, and therapeutic products has attracted significant attention due to technical advantages like stability under varying pH and thermal conditions, which also contribute to lower production and storage costs [283]. Market trends suggest increasing innovation from industry stakeholders in developing postbiotic-based formulations, such as CytoFlora^®^ and Del-Immune V^®^, which highlight the commercial viability of these compounds [283]. However, a major hurdle for industrial scalability remains the absence of clear regulatory standards and definitions, which hampers consistent quality control and product classification [37]. Current global regulations do not provide a unified framework for postbiotic approval, which complicates claims, labelling, and product development [37,285]. To ensure safe and standardised commercialisation, future efforts must focus on establishing regulatory definitions, validated health claims, production standards, and appropriate delivery systems for specific health conditions. Addressing these gaps will help translate the scientific promise of postbiotics into sustainable economic and therapeutic outcomes [283].

## 8. Challenges and Future Directions in Postbiotic Research and Therapeutic Applications

This review highlighted the potential therapeutic applications of postbiotics due to their pleiotropic effects, including antioxidant, anti-inflammatory, and immunomodulating activities, as well as their direct influence of gut barrier tightness, intestinal ecosystems, and shaping the gut bacterial signature. Researchers have begun to identify specific postbiotic components that significantly interact with human metabolic and physiological processes to promote healthy ageing, health-modifying impacts, and longevity. However, postbiotics also face challenges and limitations that need to be clearly addressed to realise their full therapeutic applications and enhance patient compliance. Despite breakthroughs demonstrated in both preclinical and clinical investigations, some studies have blurred the line between prebiotics and postbiotics, as their impacts were often not separately assessed [122].

Another research limitation is related to translating findings due to physiological differences between the tested animal models and humans in terms of gut microbiome signatures, immune signalling pathways, and environmental covariates [286]. This can complicate the accurate mimicry of age-related conditions, hinder outcome generalisation and challenges against robust clinical recommendations. A further challenge in using postbiotics for specific medical conditions is the knowledge gap regarding their health-modifying effects mechanisms. Most highlighted postbiotics mechanisms are associated with modulating the animal gut-brain axis; however, more research is needed to broaden our understanding of their interlink with human body/mechanisms for managing diseases [122].

Inconclusive or even contradictory study outcomes have been noted due to the inherit postbiotic characteristics and study design variability [286]. Some investigated postbiotics possess immunogenic properties like those of the Gram-negative and Gram-positive cell wall components, such as lipopolysaccharides and lipoteichoic acids, respectively. While some studies demonstrated anti-inflammatory activities of lipoteichoic acid through reduced IL-12 and boosted IL-10 productions [287], others found a lack of significant anti-inflammatory activity for this cell wall component, which can cause gut membrane injury [288]. This discrepancy may be due to non-standardised postbiotic preparation methods, derived probiotic strains, administration route, dosages, and formulations across studies, complicating comparisons and identification of optimal usage approaches [289]. Although postbiotics offer promising functional and formulation advantages, unresolved knowledge gaps regarding their precise composition continue to limit their safe application and hinder their classification as pharmaceutical agents with consistent active components. As a result, they are more commonly developed as food supplements or incorporated into functional foods, such as yoghurts [290].

A unique limitation of postbiotics is that they cannot proliferate or grow within the gut and do not impose sustained effects after supplementations is discontinued. Thus, innovative delivery systems are needed to extend their effectiveness and reduce administration frequency [291]. Research studies can also rely on quantitative estimation of novel postbiotic biomarkers correlated with positive health impacts. Quantifying postbiotic biomarkers can help determine whether a host microbiome biolevel is insufficient or indicative of a medical condition and whether postbiotic supplementation would be beneficial [292].

Owing to their demonstrated health benefits, postbiotics are being explored for integration into food and health-related products. There are some postbiotic products are currently available for human usage in the markets [61,62,170]. Nevertheless, challenges for postbiotics food supplementations arise from the regulatory and standardisation hurdles. The absence of consistent classification frameworks poses challenges to standardisation and safety assurance of postbiotic-based interventions [293]. Individual variabilities in terms of microbiome signatures, dietary patterns, epigenetics, as well as patient compliance related to taste, cost, and convenience, can pose challenges and require more holistic understanding and extensive research. Additionally, consumption of certain postbiotics can be conflicting with certain religions or cultural beliefs, posing challenges for market access, product registration, and development [101]. These challenges pave the way for future research directions focusing on personalised postbiotic utilisation tailored to individual microbiome signatures and health states [294]. Moreover, ethical best practices in terms of interdisciplinary collaborations, long-term clinical trials, and strategies for affordability and accessibility must be considered, as postbiotics represent the intersection of nutrition, therapeutics, and ecological sciences.

## Figures and Tables

**Table 1:** Selected studies highlighting the antioxidant capacities of postbiotics.

Postbiotics Component	Isolation Characteristics from Probiotic Strain	Key Study Findings	Mechanistic Aspects	References
Heat-treated, non-viable	Heat-treated *Bifidobacterium longum* CECT-7347 by autoclaving for 20 min at 121 °C, 1 atm pressure.	Increased Caenorhabditis elegans survival rates after oxidative stress.	Activated DAF-16 (worm homolog of FOXO transcription factor), decreased IL-8, and suppressed NF-κB signalling.	[145]
Cell-free supernatant	*Lactiplantibacillus plantarum* RI11 grown in MRS broth at 37 °C for 48 h.	Increased IL-10; reduced IL-8, HSP70, TNF-α, and α1-acid glycoprotein.	Elevated serum glutathione peroxidase and Zn/Cu superoxide dismutase levels.	[146]
	*L. plantarum* RG11, RG14, and TL1 cultured in MRS broth at 30 °C for 10 h.	Improved antioxidant activity and regulation of rumen barrier function in postbiotic-treated animals.	Elevated serum glutathione peroxidases and Zn/Cu superoxide dismutases.	[147]
	*L. plantarum* SN4 and *Bacillus amyloliquefaciens* J cultured in LB or MRS broth at 37 °C for 10 h.	Demonstrated broad-spectrum antibacterial effects, strong antioxidant activity, anti-inflammatory effects, and intestinal wound healing.	Inhibited nitric oxide (NO) production.	[148]
	*Lactobacillus* spp. (*L. acidophilus*, *L. casei*, *L. lactis*, *L. reuteri*), and *Saccharomyces boulardii* cultured in RPMI 1640 at 37 °C for 24 h.	Reduced oxidative damage and exhibited strong free radical scavenging.	Downregulated PGE-2, IL-8, IL-1β, IL-6, TNF-α; upregulated IL-10 production by human macrophages.	[149]
Exopolysaccharides	*Lactobacillus fermentum* S1 grown in fermentation broth at 37 °C for 10 h under aerobic conditions; proteins removed with trichloroacetic acid.	Exhibited in vitro antioxidant activity against free radicals.		[110]
	*Paenibacillus mucilaginosus* TKU032 grown in SPP-treated medium at 37 °C for 6 days aerobically with agitation; exopolysaccharides precipitated with ethanol.	Demonstrated in vitro antioxidant activity and reactive oxygen species (ROS) scavenging.		[150]
	*L. helveticus* MB2-1 cultured in fermentation broth at 33 °C for 24 h aerobically; precipitated with 0.9% NaCl, sonicated, and reprecipitated with 75% ethanol.	Showed in vitro antioxidant and ROS scavenging activity; enhanced total antioxidant capacity and superoxide dismutase (SOD) activity, and reduced malondialdehyde in rats.	Activity attributed to uronic acid polysaccharide binding ferrous iron, similar to green tea.	[151,152]
Cellular isolates	*Leuconostoc pseudomesenteroides* processed via enzymatic lysis and sonication.	Exhibited in vitro antioxidant and ROS scavenging activity.	Demonstrated higher antioxidant activity than controls.	[153]
Lysates	*L. casei* CRL431 lysed via enzymatic treatment and sonication.	Exhibited in vitro antioxidant and ROS scavenging activity.	Modulated antioxidant response under aflatoxin B1-induced oxidative stress.	[154]

**Table 2:** Selected studies highlighting the postbiotics’ anti-inflammatory and immunomodulation properties.

Postbiotics Component	Isolation Characteristics from Probiotic Strains	Key Study Findings	Mechanistic Aspects	References
Lipoteichoic acid	Extracted from *Lactobacillus rhamnosus* using butanol/phenol-based solvent from cell-free supernatant.	Reversed UV-induced immunosuppression in skin hypersensitivity.	Activated dendritic cells and T-lymphocytes in the skin.	[167]
Peptidoglycans	Lipid removed from *L. rhamnosus* via organic solvent extraction following sonication to disrupt the cell wall.	Reversed polyinosinic:polycytidylic acid (poly I:C)-induced lung injuries.	Increased IL-α, IL-β, IL-γ, IL-6, and IL-10 levels via TLR-3 activation.	[168]
Exopolysaccharides	Extracted from *Lactobacillus delbrueckii* using protein precipitation with trichloroacetic acid followed by ethanol precipitation at 4 °C overnight.	Improved gut health and inhibited viral replication.	Balanced pro- and anti-inflammatory cytokine responses.	[169]
Short-chain fatty acids (acetate, propionate, butyrate)	Isolated via liquid-liquid extraction using water:acetonitrile, followed by vortexing and filtration.	Maintained colon health by restoring gut microbiome balance.	Activated GPCR-43, promoting beneficial bacterial growth and inhibiting pathogenic bacteria.	[75]
Bacteriocin	Harvested from *Lactococcus lactis* through cell collection and solvent extraction using chloroform.	Promoted differentiation and proliferation of B- and T-lymphocytes.	Increased IL-1β and IL-6 levels.	[170]

**Table 3:** Selected preclinical and clinical studies highlighting the postbiotics’ cardiovascular modulating activities.

Postbiotics Component	Study Description	Findings	References
Propionic acid, Butyric acid, Acetic acid	1 g/kg parenteral administration in C57BL/6 mice.	Reduced heart rate and mean arterial pressure.	[239]
	13-week oral supplementation (0.5 mg/kg/day) in spontaneously hypertensive rats.	Reduced blood pressure.	[240]
	Observational clinical cohort study of 92 patients; SCFA levels measured in faecal and blood samples and correlated with vascular calcification scores.	Inverse correlation between SCFA levels and vascular calcification and lipid profiles.	[241]
Butyric acid Acetic acid	8-week oral supplementation (68 mM acetate; 40 mM butyrate) in TLRY264H lupus-prone mice.	Enhanced endothelial-dependent vasodilation, reduced blood pressure and left ventricular hypertrophy, and improved gut barrier integrity.	[242]
Propionic acid	Oral administration (200 mmol/L) in angiotensin II-infused wild-type and ApoE–/– mice.	Decreased systolic and diastolic blood pressure.	[243]
	4-week oral supplementation (200 mg/kg) in high-fat diet-fed ApoE–/– mice.	Reduced plasma LDL, VLDL, total cholesterol, and atherosclerotic lesion size.	[244]
	8-week oral supplementation (500 mg, BID) in hypercholesterolemic patients.	Decreased plasma lipid concentrations.	
	6-week oral or rectal administration (200 mM) in a vitamin D3/nicotine-induced vascular calcification rat model.	59% reduction in aortic calcium content; decreased TNFα, IL-1β, and IL-6 expression; reduced vascular macrophage infiltration; improved gut dysbiosis and barrier integrity.	[241]
Butyric acid	IV administration (0.14–5.6 mmol/kg) in rats.	Lowered blood pressure.	[245]
	28-day parenteral administration (1 g/kg) in angiotensin II-infused mice.	Improved vasodilation response in pre-contracted aortic rings.	[246]
